# Different levels of recruitment during conduction system pacing

**DOI:** 10.1016/j.hrcr.2024.09.013

**Published:** 2024-12-16

**Authors:** Johan van Koll, Jesse H.J. Rijks, Kevin Vernooy, Jacqueline Joza

**Affiliations:** 1 Department of Cardiology, Cardiovascular Research Institute Maastricht, Maastricht University Medical Centre, Maastricht, The Netherlands; 2 McGill University Health Center, Montreal, Québec, Canada

**Keywords:** Left bundle branch area pacing, His Bundle pacing, Conduction system pacing, ECG-analysis, Right Bundle Branch Block, Case report

## Introduction

His bundle pacing (HBP) and left bundle branch pacing (LBBP) are forms of conduction system pacing (CSP) that aim to preserve normal physiologic ventricular activation.^1^ This is especially preferred in patients with an anticipated high ventricular pacing percentage to mitigate the risk of pacing-mediated cardiomyopathy from traditional right ventricular pacing (RVP).^2^^,^^3^ Both forms of CSP have been shown to be effective in maintaining physiologic ventricular activation with improvement in clinical outcomes.^4^ Although both forms capture the conduction system, LBBP is associated with lower capture thresholds and higher implant success rates when compared with HBP.^5^ During implantation, careful evaluation of the 12-lead ECG is necessary to differentiate between different levels of conduction system capture.^6^ Nevertheless, ECG interpretation can be challenging, requiring awareness of potential pitfalls.

In this report we describe a CSP case in the presence of temporary right bundle branch block (RBBB), where differentiating between various degrees of conduction system capture proved to be particularly challenging.

**Test your knowledge!**

• Test your knowledge! Take an interactive quiz related to this article: https://www.heartrhythmcasereports.com/content/quiz_archive

## Case report

A 75-year-old woman with symptomatic persistent atrial fibrillation was referred for permanent pacemaker implantation with LBBP and atrioventricular node ablation. The preimplantation ECG showed atrial fibrillation with a ventricular response rate of ±100 bpm, and a narrow QRS duration of 98 ms.

### Lead implantation

**Figure 1:**
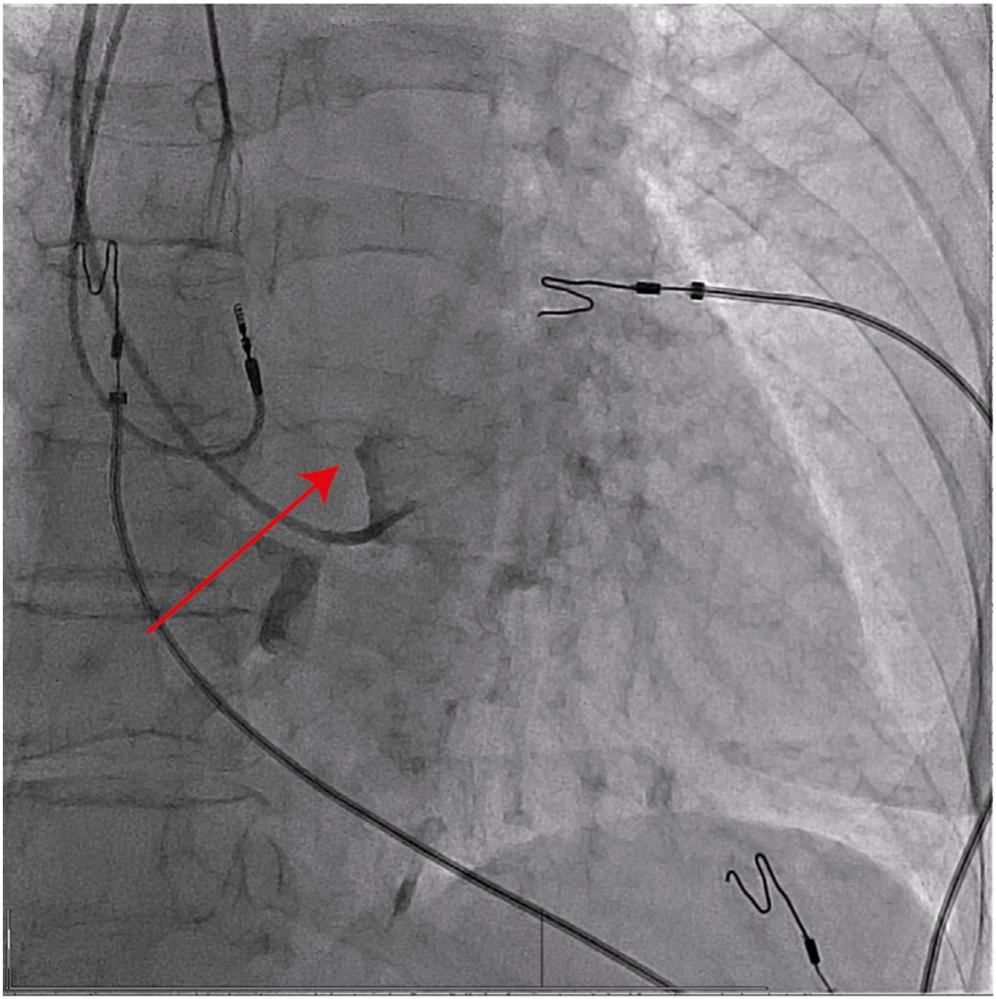
Fluoroscopy-guided right anterior oblique contrast injection in the right ventricle using the C315HIS to identify the region of the bundle of His. The *red arrow* indicates the His bundle region.

**Figure 2:**
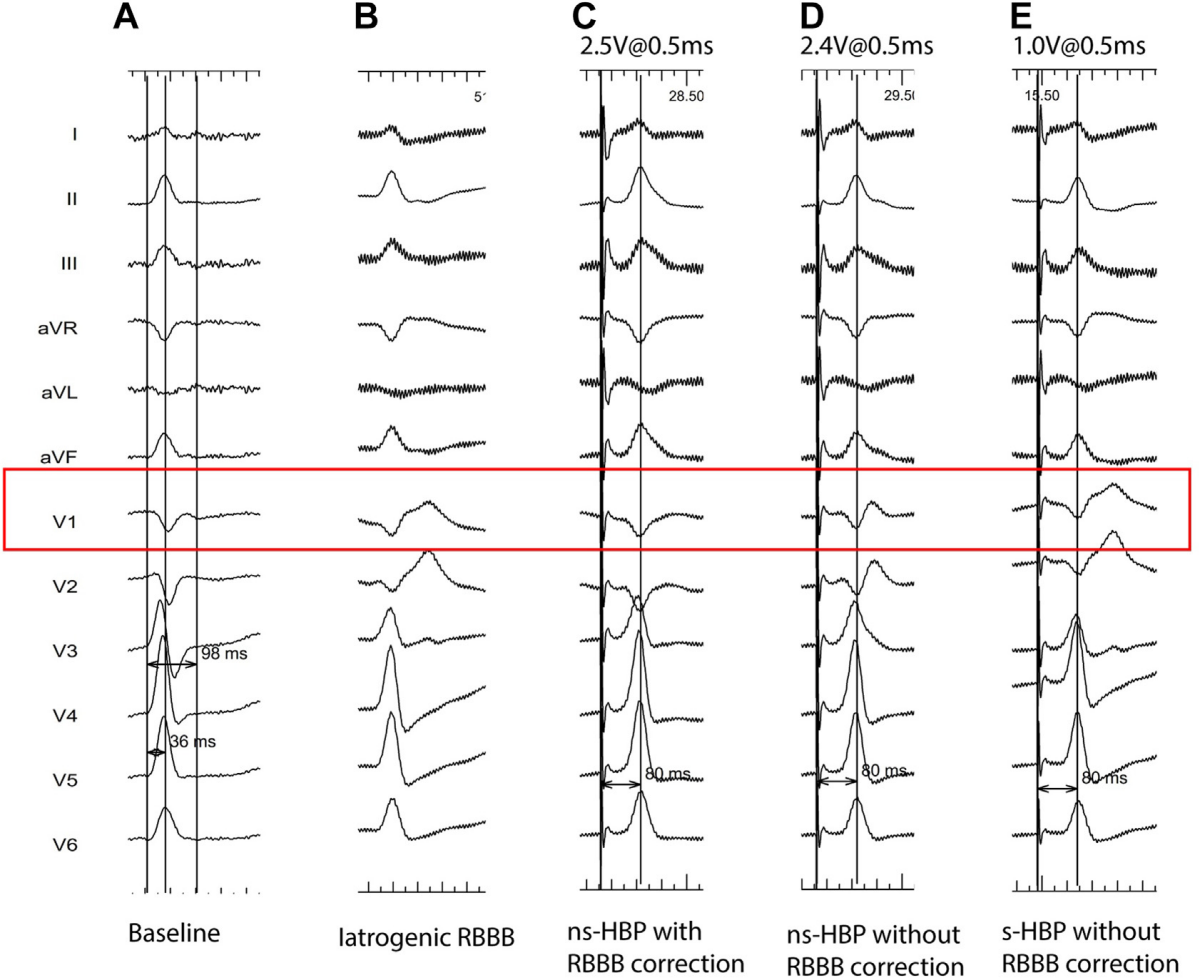
Twelve-lead electrocardiograms (ECGs) at different outputs during left bundle branch pacing (LBBP) lead implantation. **A:** Twelve-lead ECG at baseline. **B:** Twelve-lead ECG of the iatrogenic/intrinsic right bundle branch block (RBBB). **C:** Twelve-lead ECG at a pacing output of 2.5 V at 0.5 ms. **D:** Twelve-lead ECG at a pacing output of 2.4 V at 0.5 ms. **E:** Twelve-lead ECG at a pacing output of 1.0 V at 0.5 ms. The V6 R wave peak time (V6RWPT) is presented in all paced ECGs.

Lead implantation was performed as described previously.^2^ In short, the region of the bundle of His was identified using a fluoroscopy-guided contrast injection in the right ventricle (RV) (Figure 1). The C315His (Medtronic Inc, Minneapolis, MN) guiding sheath was advanced 1–1.5 cm from the His bundle region toward the RV apex. During maneuvering of the guiding sheath within the RV, iatrogenic RBBB was induced (Figure 2B). Temporary iatrogenic RBBB is a phenomenon that is observed frequently with catheter manipulation in the RV in up to 20% of cases.^7^ When the optimal initial location for proximal LBBP was identified, the lead was then advanced into the interventricular septum. No His bundle or left bundle branch potentials were noted before or during lead fixation.

### Pacing and conduction assessment

After lead deployment, decremental output pacing was performed to assess conduction system capture (Figure 2). During high-output unipolar pacing, a QRS morphology was observed that resembled the initial narrow QRS, before RBBB creation. At this high output, the paced V6 RWPT (time from pacing spike to peak R-wave in lead V6) was measured at 80 ms, with a remarkably narrow QS pattern and a pseudo delta-wave consistent with nonselective His bundle pacing (ns-HBP; Figure 2C). It is here that the right bundle (RB), left bundle (LB), and myocardium are captured.

With reduction in the pacing output to 2.4 V at 0.5 ms, a QRS morphology transition was observed from a narrow QS to a qR pattern in lead V1 and an unchanged V6 RWPT (Figure 2D). This indicates capture of the LB and the myocardium with loss of RB capture. The complete RBBB pattern is not entirely present because of persistent septal myocardial capture.

A further decrease in pacing output to 1.0 V at 0.5 ms reveals a third QRS morphology transition to a complete RBBB pattern (Figure 2E), resembling the underlying iatrogenic RBBB QRS pattern (Figure 2B), with a consistent V6RWPT of 80 ms. In this situation, there appears to be loss of septal myocardial capture and conduction only via the LB.

### Follow-up

Six weeks after device implantation, the atrioventricular node ablation was performed, and a transthoracic echocardiogram was performed. No adverse clinical outcomes were noted until the 6-month follow-up.

## Discussion

In this case-report, we describe a patient who underwent permanent pacemaker implantation as part of a pace-and-ablate strategy with the aim of LBBP. During implantation, several QRS morphology transitions were obtained, where recruitment of various parts of the conduction system is sequentially lost with decremental pacing output. In the present case, three distinct QRS morphologies were noted during unipolar pacing with decremental output: (1) fully narrow QRS during high-output pacing, (2) qR pattern in lead V1 during reduction in output, and (3) typical RBBB morphology in V1. Importantly, the V6 RWPT was maintained throughout each transition suggesting consistent capture of the LB.

Successful implantation of an LBBP-lead usually results in an R/r′ pattern in V1 during unipolar pacing except in rare circumstances.^8^ During progression of the lead from the right septum to the left endocardial septum, the QRS will become narrower as it progresses through stages of deep septal (wide, notched with left bundle branch block [LBBB] pattern in V1), left ventricular septal (LVSP) (r′ present), and finally LBB capture where a Qr/qR/rsR′ QRS pattern appears in lead V1, typically with an isoelectric interval and a shorter V6 RWPT than seen in LVSP.^9^ An rsR′ is more specific for selective LBBP (s-LBBP).^9^, ^10^, ^11^, ^12^

**Figure 3:**
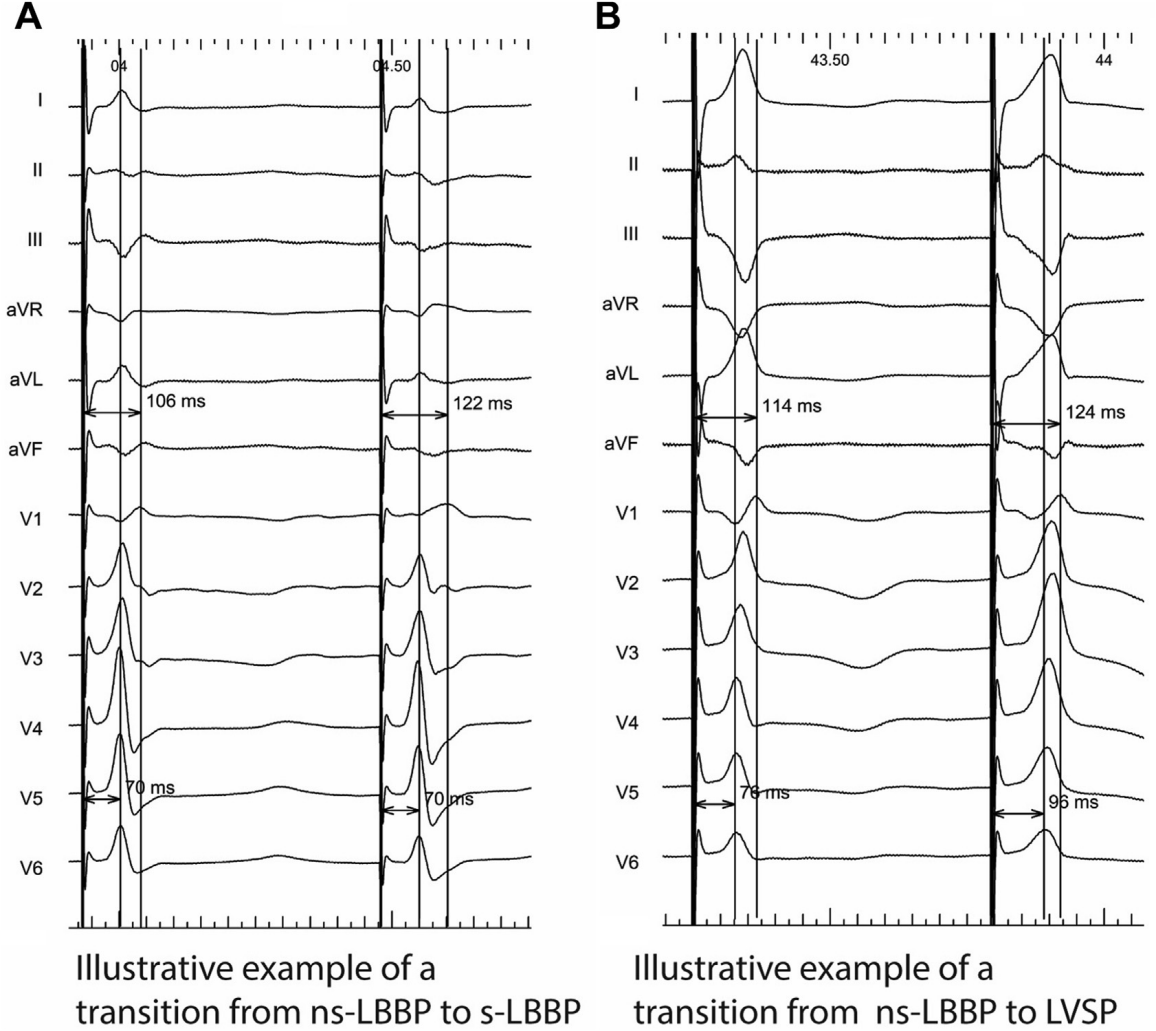
Illustrative example of (**A**) transition from ns-LBBP to s-LBBP, with a stable V6RWPT and prolongation of the QRS (and V1RWPT) and (**B**) transition from ns-LBBP to LVSP, with a prolongation of V6RWPT.

In cases of nonselective LBBP (ns-LBBP), decremental voltage output pacing should result in a transition to either s-LBBP or only LVSP.^9^ A transition from ns-LBBP to s-LBBP is characterized by a stable paced V6RWPT and a prolongation of the QRS, usually with subtle changes in QRS morphology reflecting the absence of septal myocardial capture and therefore a longer time to activate the right ventricle (Figure 3A).^9^ Conversely, a transition from ns-LBBP to LVSP is characterized by a prolongation in paced V6RWPT with a reduction in the amplitude of the *R* or *r* with disappearance of the S-wave in I/V6 as a result of loss of capture of the conduction system, resulting myocardial capture only (Figure 3B).^9^ In the case of HBP, another transition can be observed. Ns-HBP is characterized by depolarization immediately after the pacing stimulus, resulting in a pseudo–delta wave. Decremental output pacing can result in a transition to selective-HBP (s-HBP), characterized by narrowing of the QRS duration with the development of an isoelectric interval between pacing stimulus and QRS onset.^9^

Previous studies have shown that HBP can correct an LBBB.^13^ To correct an LBBB, a higher pacing output is usually needed when pacing proximal in the His bundle, as compared with pacing distal in the His bundle. The iatrogenic RBBB, as observed in this patient, is most likely distal to the His bundle. Mahmud et al.^14^ have shown that HBP at higher outputs can partially correct both an acute and chronic RBBB, even when located distal to the site of pacing. At these higher pacing outputs, ns-HBP is usually obtained.

**Figure 4:**
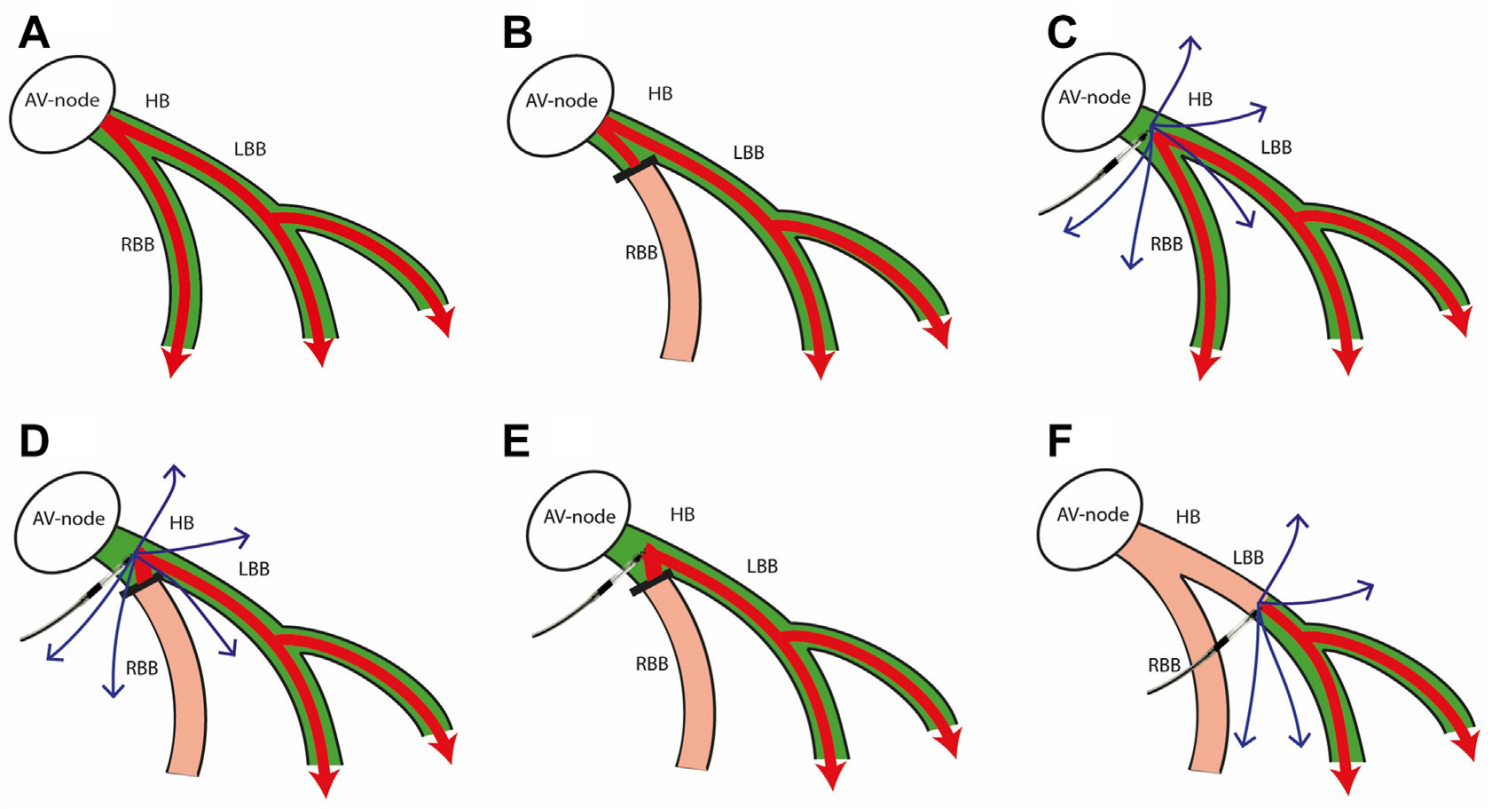
Schematic overview of different capture forms, corresponding with electrocardiogram patterns during decremental output pacing observed in this patient, per Figure 2. **A:** Intrinsic conduction without bundle branch blocks. **B:** Intrinsic conduction with (iatrogenic) right bundle branch block. **C:** Nonselective His bundle pacing with correction of right bundle branch block. **D:** Nonselective His bundle pacing without correction of right bundle branch block. **E:** Selective His bundle pacing without correction of right bundle branch block. **F:** Example of nonselective left bundle branch area pacing. *Blue arrows* indicate stimulation fronts to surrounding myocardium. AV = atrioventricular; HB = His bundle, LBB = left bundle branch; RBB = right bundle branch.

As a result, the capture of the distal His bundle was considered in the present case. During high output pacing (>2.5 V), the paced QRS morphology (Figure 2C) closely resembles the narrow QRS morphology observed before RBBB was induced (Figure 2A). This high pacing output (>2.5 V) results in ns-HBP with correction of the iatrogenic RBBB (Figure 4C), explaining why the QRS morphology is comparable to the QRS morphology at baseline. Lowering the pacing output (<2.5 V at 0.5 ms; Figure 2D) resulted in ns-HBP without correction of the iatrogenic RBBB (Figure 4D), thereby mimicking the QRS morphology (especially in lead V1) of ns-LBBP (Figure 4F). In case of ns-HBP with an (iatrogenic) RBBB, the bundle of His is activated with conduction only through the LB, mimicking LBBP. Further decrease of the pacing output led to s-HBP (Figures 2E and 4E) revealing a QRS morphology comparable to the QRS morphology with iatrogenic RBBB (Figures 2B and 4B). In the latter, only the conduction system is captured, which because of the underlying RBBB results in conduction through the LB only. In summary, the transitions in QRS morphology observed during this procedure are ns-HBP with RBBB correction to ns-HBP without RBBB correction, to finally s-HBP with underlying RBBB.

**Figure 5:**
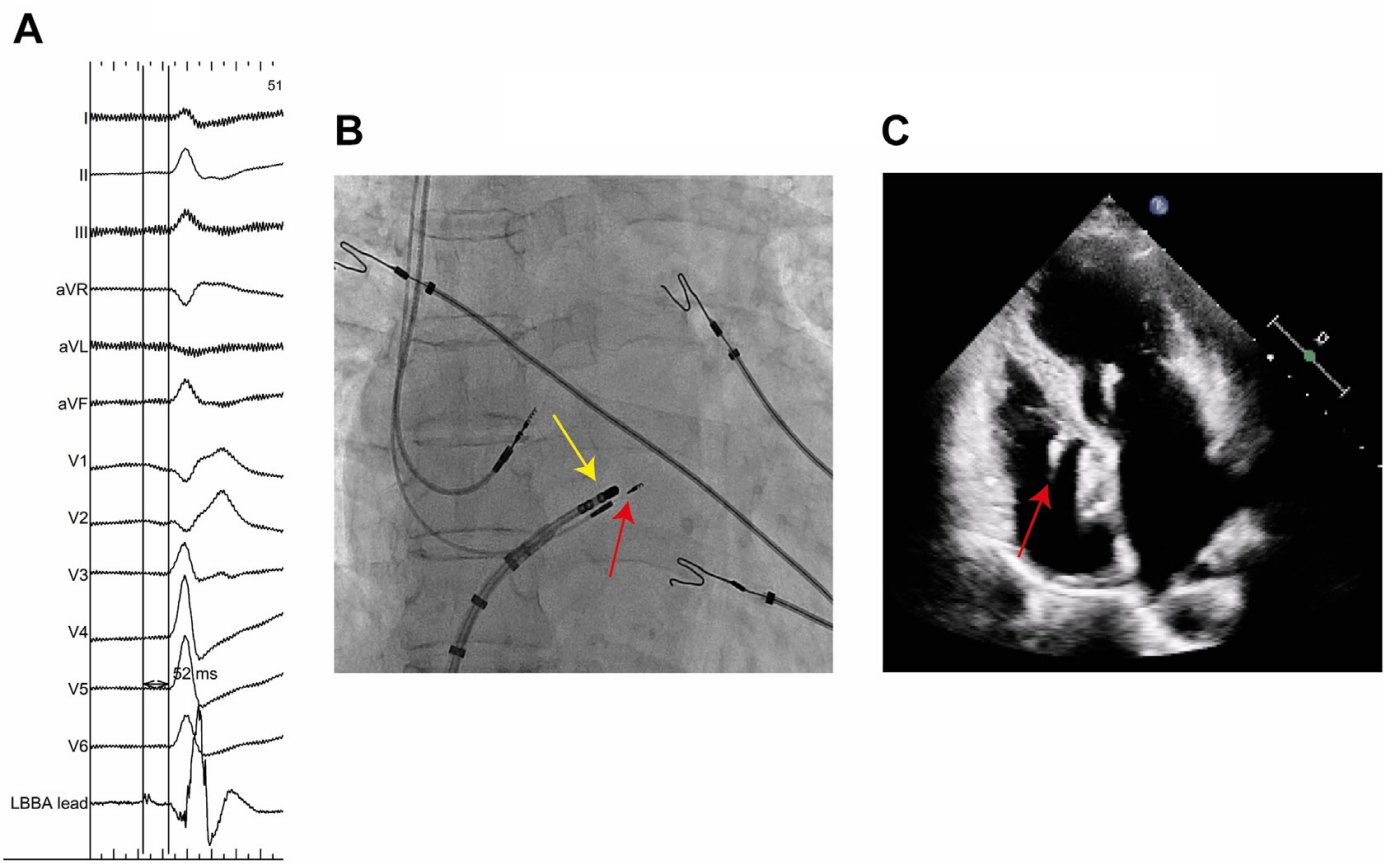
**A:** Twelve-lead electrocardiogram after lead implantation, including local electrocardiogram: His bundle potential to QRS onset interval of 52 ms. **B:** Ablation catheter during His bundle ablation in proximity of implanted pacing lead. The *yellow arrow* indicates the ablation catheter, and the *red arrow* indicates the implanted pacing lead. **C:** Apical four-chamber view of transthoracic echocardiogram at follow-up. The *red arrow* indicates the implanted pacing lead. LBBA = left bundle branch area.

**Figure 6:**
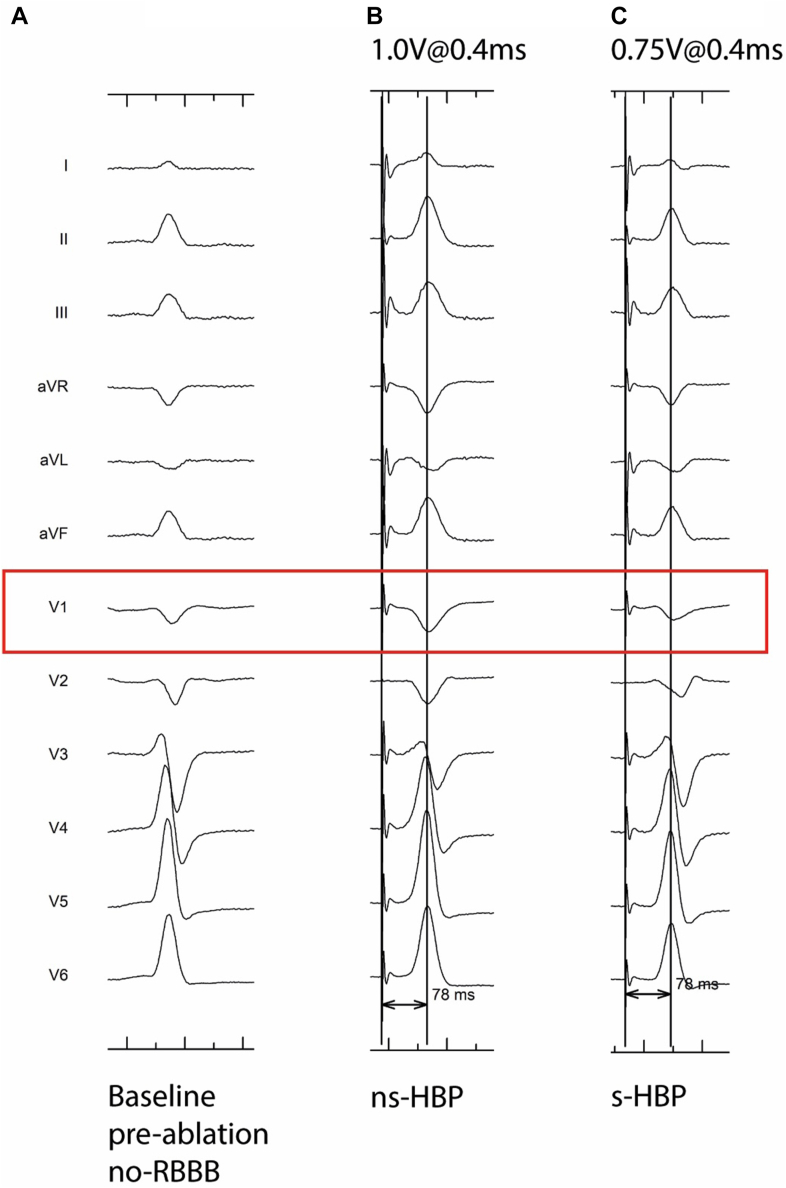
Follow-up 12-lead electrocardiograms (ECGs) 6 weeks after implantation. **A:** Intrinsic ECG before His bundle ablation, no right bundle branch block (RBBB). **B:** Twelve-lead ECG at a pacing output of 1.0 V at 0.4 ms. **C:** Twelve-lead ECG at a pacing output of 0.75 V at 0.4 ms.

In addition to the different transitions observed, the His bundle potential to QRS-onset interval of 52 ms (Figure 5A) at the final pacing location further suggests HBP in this case.^9^ Follow-up data further confirm the capture of the distal His bundle. A radiograph obtained during His bundle ablation shows the proximity of the ablation catheter (proximal) to the implanted pacing lead, suggesting a close positioning of the pacing lead to the His bundle (Figure 5B). In addition, the follow-up transthoracic echocardiogram displays the basal position of the pacing lead (Figure 5C). This was again in line with the understanding that we obtained HBP instead of the planned LBBP in this patient. Furthermore, follow-up showed recovery of normal conduction with resolution of the RBBB, and during decremental output pacing, a paced narrow QRS comparable to normal intrinsic conduction was obtained at all pacing outputs. In addition, a transition from ns-HBP to s-HBP was found to be present (Figure 6), further confirming capture of the distal bundle of His in this case.

### Comparable QRS morphologies during different conduction patterns

Figure 4 schematically demonstrates regional conduction capture corresponding with the findings observed in this case report. The conduction patterns noted in Figure 4A and 4C are highly comparable, suggesting a similar QRS morphology (as in Figure 2A and 2C)—that is, QRS morphology of intrinsic conduction resembles that of ns-HBP with RBBB correction in this case. Figure 4C shows ns-HBP where the pacing output is high enough to correct the iatrogenic RBBB (thus conduction through the LB, RB, and local myocardium), leading to comparable QRS morphology as the baseline. The conduction pattern noted in Figure 4E resembles that of Figure 4B, where the QRS morphology in s-HBP is comparable to the iatrogenic RBBB in this case. In case of s-HBP in the presence of an RBBB, the His bundle is captured with conduction only through the LB, resulting in comparable QRS morphology to the underlying iatrogenic RBBB. Lastly, during this procedure, the conduction in Figure 4D is comparable to the conduction in Figure 4F, resulting in comparable QRS morphology in ns-HBP without correction of RBBB and ns-LBBP (Figure 2C and 2F). In case of ns-HBP without correction of RBBB, the LB and myocardium are captured, comparable to ns-LBBP, resulting in comparable QRS morphology.

## Conclusion

It is commonly mentioned that careful analysis of the ECG is important during the implantation of a CSP lead, where different potential pitfalls complicate the interpretation.^6^ This case report underscores the sometimes challenging ECG interpretation during CSP implantation and emphasizes the importance of understanding its complexity. We describe an attempt to implant an LBBP lead, during which an iatrogenic RBBB was induced, complicating ECG interpretation. Careful ECG evaluation revealed capture of the distal His bundle with both ns-HBP and s-HBP at different output levels, and correction of the iatrogenic RBBB at higher outputs. This case illustrates that QRS morphology during ns-HBP without correction of the iatrogenic RBBB can mimic the QRS morphology of ns-LBBP. However, accurately identifying the His bundle location prior to LBBP lead implantation could have helped identify the mechanism of QRS morphology changes during intended LBBP in this case.

In LBBP, the qR/Qr/rSR’ pattern in lead V1 may be rarely masked despite LBB capture.^8^ This phenomenon could result from almost simultaneous capture of the proximal LB with the RB at high output pacing.^8^ Although this potential pitfall is rarely encountered, it highlights the importance of understanding the complexity of CSP.

## Disclosures

Kevin Vernooy receives research and educational grants and has consultancy agreements with Medtronic, Abbott, Boston Scientific, Philips, and Biosense Webster (all grants are paid to the institute). Jacqueline Joza receives a research grant from Medtronic and is a consultant for Boston Scientific, Abbott, and Medtronic. The rest of the authors have no conflicts of interest.
